# Epigenetic Modification Mechanisms Involved in Inflammation and Fibrosis in Renal Pathology

**DOI:** 10.1155/2018/2931049

**Published:** 2018-12-13

**Authors:** Jose Luis Morgado-Pascual, Vanessa Marchant, Raul Rodrigues-Diez, Nuria Dolade, Beatriz Suarez-Alvarez, Bredford Kerr, Jose M. Valdivielso, Marta Ruiz-Ortega, Sandra Rayego-Mateos

**Affiliations:** ^1^Cellular Biology in Renal Diseases Laboratory, Universidad Autónoma Madrid, IIS-Fundación Jiménez Díaz, Madrid, Spain; ^2^RedInRen RETIC, ISCIII, Spain; ^3^Centro de Estudios Científicos (CECs), Valdivia 5110466, Chile; ^4^Faculty of Medicine, Universidad Austral de Chile, Valdivia 5090000, Chile; ^5^Nephrology Department, IDIPAZ, Madrid, Spain; ^6^Vascular and Renal Translational Research Group, Institut de Recerca Biomèdica de Lleida IRBLleida, Lleida 25198, Spain; ^7^Translational Immunology Laboratory, Health Research Institute of the Principality of Asturias (ISPA), Hospital Universitario Central de Asturias, Oviedo, Spain

## Abstract

The growing incidence of obesity, hypertension, and diabetes, coupled with the aging of the population, is increasing the prevalence of renal diseases in our society. Chronic kidney disease (CKD) is characterized by persistent inflammation, fibrosis, and loss of renal function leading to end-stage renal disease. Nowadays, CKD treatment has limited effectiveness underscoring the importance of the development of innovative therapeutic options. Recent studies have identified how epigenetic modifications participate in the susceptibility to CKD and have explained how the environment interacts with the renal cell epigenome to contribute to renal damage. Epigenetic mechanisms regulate critical processes involved in gene regulation and downstream cellular responses. The most relevant epigenetic modifications that play a critical role in renal damage include DNA methylation, histone modifications, and changes in miRNA levels. Importantly, these epigenetic modifications are reversible and, therefore, a source of potential therapeutic targets. Here, we will explain how epigenetic mechanisms may regulate essential processes involved in renal pathology and highlight some possible epigenetic therapeutic strategies for CKD treatment.

## 1. Introduction

**Figure 1:**
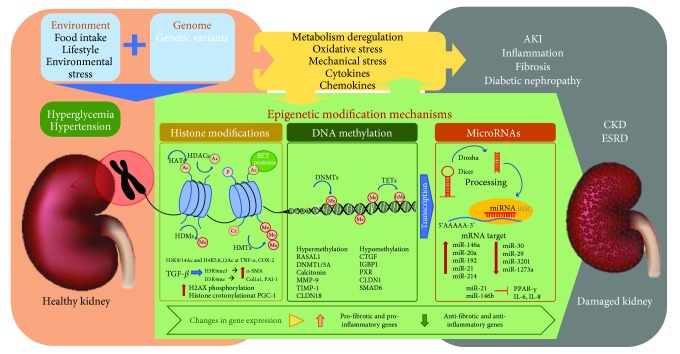
The main epigenetic changes associated with clinical and experimental renal damage such as DNA methylation (DNAme), histone posttranslational modifications, and miRNAs.

The term “epigenetics” was first used by Conrad Waddington in 1942 to describe the effect of gene-environment interactions on the expression of particular phenotypes [1–3]. Not only does gene expression depend on the different changes in the cellular machinery surrounding DNA expression but also the environment exerts a powerful influence on the gene expression. It can be said that epigenetic changes influence the gene expression, but it is only a part of the variability that exists in a cell when it comes to expressing information contained in DNA. Epigenetic mechanisms are fundamental for normal development and maintenance of tissue-specific gene expression through changes on chromatin structure, function regulation, and posttranscriptional mechanisms. These epigenetic modifications include DNA methylation (cytosine methylation), histone posttranslational modifications, and miRNAs (Figure 1) [4].

Epigenetics has recently been shown to have impact on human health and disease susceptibility. Although there is a genetic predisposition for the development of chronic kidney disease (CKD), genetics alone falls short of explaining the complex disease progression across individual CKD patients [5]. Many factors altered in CKD patients, such as uremic toxins, oxidative stress, and inflammation, as well as changes in metabolic states, like hyperglycemia in diabetes, can induce epigenetic modifications by regulating gene expression and altering the immune response and therefore contribute to renal disease progression [6]. The identification of these epigenetic changes is a recent and innovative field of research in renal pathology [7]. In this review, we summarize the epigenetic mechanisms that could regulate critical processes in renal pathology, including inflammation and extracellular matrix accumulation (ECM), and offer an account of potential therapeutic strategies based on epigenetic targets for CKD treatment (Figure 1).

### 1.1. DNA Methylation and Kidney Diseases

DNA methylation is a common epigenetic mechanism used by cells to express or silence a gene [8]. DNA methylation mainly occurs on the cytosine residues of CpG (cytosine-phosphate-guanine) sites [9], and in mammalians, 60-90% of them are methylated [10]. DNA methylation patterns are specific of tissue/cell type maintaining gene expression states [11]. Thus, as a paradigm, hypomethylated DNA is associated with active transcription, whereas hypermethylated DNA is packaged in inactive chromatin (heterochromatin) [12].

DNA methylation is catalyzed by a family of enzymes named DNA methyltransferases (DNMTs), which transfer a methyl group from S-adenosyl methionine (SAM) to the fifth carbon of cytosine at the dinucleotide CpG. DNMTs can be classified in two groups: (1) maintenance DNMTs such as DNMT1, which shows a preference for hemimethylated DNA and functions during DNA replication, and (2) de novo DNMTs including DNMT3A and DNMT3B, which methylate DNA (unmethylated or methylated) at an equal rate [13–15]. In addition, DNA demethylation can occur passively or as a result of the action of enzyme dioxygenases from the TET protein family (TET1, TET2, and TET3) that generate sequential DNA methylation cytosine changes, such as 5-hydroxymethylcytosine (5hmC), 5-formylcytosine (5fC), and 5-carboxylcytosine (5caC), culminating in excision by base-excision repair of glycosylases such as TDGs (thymine DNA glycosylases), followed by DNA repair to replace the modified cytosines by nonmethylated cytosines [16].

In the pathological context, aberrant DNA methylation and consequent deregulated gene expression could be involved with the pathogenesis of many diseases, including cancer, cardiovascular pathologies, metabolic disorders (obesity and diabetes mellitus), or renal diseases [17–21]. In the last years, several studies carried out in CKD patients revealed that systemic DNA methylation patterns correlate with the rate of kidney function decline [22, 23].

A recent epigenome-wide association study (EGWAS) in whole-blood DNA of patients from Atherosclerosis Risk in Communities (ARIC) and Framingham Heart (FHS) studies, analyzed using regression models with residualized methylation *β*-values as the independent variable, showed a statistically significant association between the DNA methylation level in specific CpG sites (e.g., cg23597162 in *JAZF1*; cg19942083 *in PTPN6/PHB2*; and cg17944885 in *ZNF788/ZNF20*) and the reduction in glomerular filtration rate (eGFR), prevalence and incidence of CKD (cg17944885 and cg19942083 in *PTPN6*/*PHB2*), or renal fibrosis (cg12065228 in *PQLC2*, intergenic cg19942083 near *PTPN6*/*PHB2*, cg12116137 in *PRPF8*, cg09022230 in *TNRC18*, and cg27660627 in *ANKRD11*). Moreover, they also found a significant enrichment of eGFR-associated CpGs in regions that bind the transcription factors (TFs), like EBF1, EP300, and CEBPB [24]. Accordingly, DNA methylation in specific CpG sites was associated with eGFR decline and fibrosis in diabetic American Indians [25].

DNA methylation patterns in specific genes could be relevant as potential biomarkers. The connective tissue growth factor (CTGF or CCN2) is a profibrotic factor, a potential biomarker of renal damage in diabetes, and a potential therapeutic target, as demonstrated in experimental models of renal injury [26, 27]. In diabetic nephropathy (DN), demethylation of the *CTGF* gene promoter correlates with low eGFR [28, 29]. *MTHFR* DNA methylation levels were higher in end-stage renal disease (ESRD) patients than in controls and associated with a decreased eGFR [30]. A study carried out in patients with type 1 diabetes (T1D) showed a decrease in *IGFBP1* DNA levels from peripheral blood samples compared to normal glucose tolerance subjects, which could be associated with the increased circulating levels of IGFBP-1 in those patients [31].

Most of the knowledge about DNA methylation on renal damage came from studies in DN. Experimental studies evaluating tubular cells of diabetic mice have found an aberrant DNA hypomethylation in genes involved in glucose metabolism, such as *Agt* (a marker of renal damage in diabetes) and *Hnf4a* (a transcription factor regulating transporters for reabsorption). In contrast, other genes were hypermethylated, such as *cldn18* [32]. Moreover, increased expression of the *pregnane X receptor* caused by aberrant demethylation of its promoter was also described [33], although the biological meaning of these findings remains to be determined. Studies carried out in streptozotocin- (STZ-) induced diabetic mice showed hypomethylation of *cldn1-*CpG regions and *Sirt1* overexpression leading to higher methylation levels of these regions. In parallel with these epigenetic changes, the levels of *cldn1* mRNA expression were lower in parietal epithelial cells from diabetic *Sirt1* transgenic mice compared to WT cells [34]. Additionally, several studies carried out in whole blood samples from DN patients have identified differentially methylated genes (*NRBF2*, *RUNX3*, *DAPK3 UNC13B*, and *DOC2A*) associated to different processes such as transcription regulation, inflammation, apoptosis, or exocytosis [35, 36]. In another study, a significant negative correlation between methylation levels of targeted genes (*TIMP-2* and *AKR1B1*) and albuminuria levels was found in early DN [37].

DNA methylation also participates in the regulation of ECM-related genes. In human kidney samples from CKD patients, differentially methylated regions located in putative enhancers have been described. Genes around these regions include *COL IVA1/2* (a key component of ECM) transforming growth factor beta (TGF-*β*) and smad proteins, with TGF-*β*/Smad being a key signalling pathway involved in ECM regulation. In the case of Smad proteins, *SMAD3* and *SMAD6* cytosine methylation changes were correlated with gene transcription levels [38]. Hyperhomocysteinemia (HHcy) is prevalent in patients with CKD and ESRD and is characterized by an abnormally high level of homocysteine in the blood. In experimental hyperhomocysteinemia-induced renal damage, DNA hypermethylation and upregulation of *dnmt1* and *dnmt3a* were followed by gene expression changes in crucial proteins involved in ECM regulation, including matrix metalloproteinase-9 (MMP-9) (an enzyme that participates in collagen degradation) and downregulation of MMP inhibitors, including tissue inhibitor of metalloproteinase- (TIMP-) 1 and -2. Besides, in injured kidneys there was an imbalance in the methylation status of *mmp9* and *timp*s, leading to increased collagen deposition. Importantly, the treatment of these animals with a DNA methylation inhibitor, 5-aza-2-deoxycytidine (5-Aza), restored matrix-degrading enzyme MMP-9/TIMP imbalance and ameliorated renal fibrosis [39]. Hypermethylation of the *rasal1* gene causes increased Ras-GTPase activity in fibroblasts, leading to proliferation and fibrosis in the kidney [40]. In the experimental model of unilateral ureteral obstruction (UUO), TGF-*β* induces suppression of Klotho, a known renal antifibrotic protein by hypermethylation of its promoter through induction of *dnmt1* and *dnmt3a* [41].

DNA methylation can also modulate the inflammatory process in the injured kidney. In dialysis patients, global DNA hypermethylation (total DNA 5-mc) in blood samples was associated with elevated inflammatory markers as ferritin and procalcitonin, the latter being a marker of inflammation due to bacterial infections [42]. In hemodialysis patients, global DNA methylation was higher than in hemodiafiltration patients and DNA methylation is most elevated in inflamed patients [43]. Experimental studies described altered DNA methylation status of a specific CpG within the IFN response element resident in the promoter region of the C3 gene in response to hypoxia and oxidative stress upon ischemia-reperfusion (I/R) injury in rats [44]. In a mouse model of I/R, the analysis of the global level of the DNA hydroxymethylation mark (5-hydroxymethylcytosine (5hmC)) assessed by immunohistochemistry and dot blot showed an apparent decrease in 5hmC levels in ischemic mouse kidney [45]. I/R injury alters the 5hmC levels at specific gene loci at proximal promoter regions of the proinflammatory genes *cxcl10* and *ifngr2* (non-ligand-binding beta chain of the gamma interferon receptor). This decrease in 5hmC levels is related to an increase in gene expression of *cxcl10 and ifngr2*. These alterations were associated with dysregulation in TET protein expression, proteins that catalyze the oxidation of 5mC into 5hmC [45]. In systemic sclerosis (SSc), a rare connective tissue disease characterized by chronic inflammation and fibrosis with deleterious effect in the kidney, DNA hypomethylation in CD4^+^ T-lymphocytes from SSc patients decreased the expression of methylation genes such as DNA methyltransferases *(DNMT)1* and methyl-CpG-binding domain proteins *(MBD)3* and *MBD4* [46, 47]. DNA methylation also participates in acute kidney injury (AKI) and renal transplantation. Methylation of the *kallikrein* promoter has been found in patients with established acute kidney injury (AKI) [48]. Additional, aberrant hypermethylation of the *calcitonin* gene promoter is more frequent in kidney transplant recipients [49].

#### 1.1.1. Demethylating Treatments

Together, these studies indicate that monitoring or targeting the epigenome could, therefore, reveal new therapeutic approaches in CKD and open up paths to biomarker discovery and targeted therapy. Demethylating agents could be useful therapeutic approaches. Currently, two demethylating drugs are in clinical use, 5-azacitidine and decitabine, for the treatment of specific forms of myelodysplastic syndrome and acute myeloid leukemia [50]. In an experimental DN model (db/db mouse), treatment with 5-azacitidine or decitabine attenuated the increased expression of *dnmt1*, markedly diminished albuminuria, and restored podocyte function [51]. Unfortunately, 5-azacitidine and decitabine have significant adverse effects because of their broad demethylating activity and cytotoxicity. Consequently, more specific and less toxic drugs are needed to elucidate the therapeutic potential of these inhibitors.

Another therapeutic experimental approach is the antihypertensive agent hydralazine, used in clinical therapy since the middle of the last century [52]. Hydralazine has been proven to attenuate renal fibrosis in a model of ischemia-reperfusion by inducing expression of hydroxylase TET3, which catalyzes hydroxymethylation and subsequently promotes demethylation of genes such as *rasal1* [53]. Other compounds presented demethylating activity, for example, Rhein, a plant-derived anthraquinone which displays strong antifibrotic properties in the experimental model of UUO, probably by reverse Klotho DNA hypermethylation [54] (Figure 1).

### 1.2. Histone Modifications and Kidney Diseases

Posttranscriptional histone modifications (PTM) play a critical role in chromatin structure acquiring two configurations, one more closed (heterochromatin) and inactive and another less compact (euchromatin) and associated with active gene transcription. These modifications include *acetylation*, *methylation*, *ubiquitination*, *propionylation* and *crotonylation* of lysine residues; *methylation*, *ribosylation* and *citrullination* of arginine residues; and the *phosphorylation and glycosylation* of serines and threonines [55]. Histone modifications can occur within the folded domain of the histones or in the N- and C-terminal domains that extend beyond the nucleosome [56, 57]. Some of these modifications exert an activating function (such as H3K4me3, AcH3, AcH4, and AcH3K9) facilitating the opening of the chromatin and the union of the transcriptional machinery, while other modifications are repressive (such as H3K27me3, H3K9me2/3, and H4K20me3,), allowing the compaction of chromatin and preventing the union of the transcriptional machinery (Figure 1).

The adding or removing of histone marks is carried out by a group of enzymes named (1) “writers,” responsible for adding (“writing”) different epigenetic marks such as HAT (histone acetyltransferase), which adds acetyl groups to histone tails, (2) “erasers,” responsible for the removal of epigenetic marks of the histone, such as histone deacetylases (HDACs) or histone demethylases (HDMs), and (3) “readers,” whose function is to recognize the different epigenetic marks added in the histones. Among the reader proteins, one of the most relevant are the BET proteins, which contain bromodomains that recognize acetylated lysine residues in the histone tails [55, 58]. Next, we will discuss how these enzymes and epigenetic modifications could participate in renal pathology.

#### 1.2.1. Histone Acetylation

Histone acetylation reduces the net positive charge of histones and weakens interactions with DNA. This perturbation in chromatin facilitates DNA transcription. The acetylation process occurs when the acetyl group (COCH_3_) is transferred from acetyl-coenzyme A (acetyl-CoA) to lysine residues, by a process regulated by histone acetyltransferases (HATs) [59]. By contrast, the acetylated residues can be recovered by three classes of histone deacetylases (HDAC): *class I HDACs* which are zinc-dependent and located in the nucleus, class II HDACs which are also zinc-dependent but are present in the nucleus and cytoplasm, and *class III HDACs* called sirtuins (Sirt 1-7) which depend upon NAD^+^ for their activity and are present in the nucleus, cytoplasm, and mitochondria [59].

**Figure 2:**
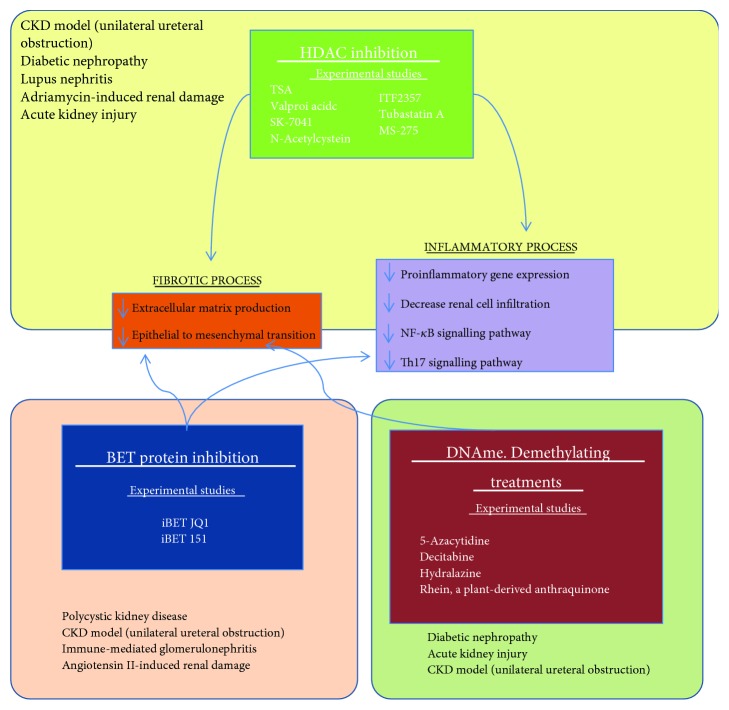
Summary of different therapeutic approaches in experimental renal damage related to DNAme and histone modifications.

Many studies have found that histone acetylation participates in experimental renal fibrosis (Figure 2). Studies done in the unilateral ureteral obstruction (UUO) model have shown that treatment with various HDAC inhibitors, such as trichostatin A or the selective class I HDAC inhibitor MS-275, reduced renal fibrosis by diminishing profibrotic markers (*α*-smooth muscle actin (SMA)) and accumulation of ECM proteins, including fibronectin and type I collagen [60–63]. In these studies, the most relevant downstream fibrotic-related mechanisms were inhibited by HDAC inhibition, including TGF-*β*/Smad, EGFR, and STAT3 signalling pathways [60] or JNK-dependent Notch-2 signalling pathways [61–63]. In experimental diabetes induced by streptozotocin injection in mice, HDAC inhibition with trichostatin A diminished profibrotic gene overexpression. Moreover, *in vitro* studies in rat tubular epithelial cells (NRK52-E cell line) showed that HDAC inhibition by the HDAC inhibitor Trichostatin A (TSA), valproic acid, SK-7041, or N-acetylcysteine or gene silencing of HDAC-1/2 restored TGF-*β*1-induced phenotypic changes, including induction of *α*-SMA and loss of epithelial markers [64], suggesting that histone acetylation could also be involved in renal fibrosis by modulating the epithelial phenotype.

HDAC inhibitors have also been involved in the regulation of renal inflammation (Figure 2). In a model of lupus nephritis, the HDAC inhibitor ITF2357 diminished the proinflammatory gene expression of *il6*, *il-1β*, and the systemic lupus erythematosus (SLE) serum biomarker IgG2a [65]. In Adriamycin-induced renal fibrosis, two HDAC inhibitors, TSA and valproic acid, reduced the expression of the chemokines *mcp1* and *mip1β* and diminished renal macrophage infiltration [66]. Similar effects of both HDAC inhibitors were described in the UUO model by reduction of CSF-1 in renal tubule interstitial space, a chemokine known to be involved in macrophage infiltration [61]. Moreover, TSA also decreased juxtaglomerular hyperplasia in damaged kidneys and the degree of fibrosis analyzed by protein levels of fibronectin and collagen I. On the other hand, TSA reduced the percentage of FOXP3^+^IL-17^+^ cells and the genic expression of *foxp3* and *rorγt*, key transcription factors involved in the modulation of Th17 differentiation [63].

Histone acetylation can also contribute to the inflammatory process associated with diabetes (Figure 2). In blood monocytes of diabetic patients, the acetylation of histones H3K9/14Ac and H4K5,8,12Ac in promoters of inflammatory genes such as TNF-*α* and COX-2 was increased [67]. In models of acute kidney injury, TSA also exerts anti-inflammatory actions, including reduction in cytokine and chemokine expression through an increase of the anti-inflammatory protein microglia/macrophage WAP domain protein [68] and a decrease in NF-*κ*B signalling [69].

There are now some ongoing clinical trials studying the effect of different HDAC inhibitors, such as belinostat, entinostat, vorinostat, and panobinostat, in renal tumors [70–72]. Previous studies in other types of tumors, including lymphoma and myeloma, have shown beneficial effects, whereas these drugs do not have enough effective activity in the treatment of renal tumors [73–75]. Thus, these data indicated that future research with epigenetic modulators in renal cancer patients would be necessary.

#### 1.2.2. Role of BET Proteins in Protein Acetylation

**Figure 3:**
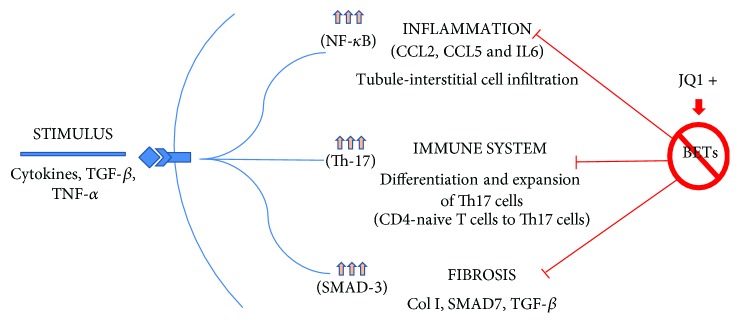
The action mechanism of the BET inhibitor JQ1, in the regulation of inflammation, Th17 response, and fibrosis.

The family of bromodomain and extraterminal (BET) proteins is composed of four members: BRD2, BRD3, BRD4 (ubiquitously expressed), and BRDT. BET proteins contain a tandem of two conserved N-terminal bromodomains (BD1 and BD2) and an extraterminal domain. BD interacts with acetylated lysine residues in histones acting as an epigenetic “reader” [76]. Studies on the cancer field have described that BRD4 contributes to the recruitment of the positive transcription elongation factor b (P-TEFb) complex (heterodimer of CDK9 and cyclin T1, T2, or K) to the promoter region and activates RNA polymerase II-dependent (RNAPII) transcription. BRD4 binds to acetylated histones in the enhancer or promoter regions of oncogenes and inflammatory genes and by this mechanism participates in malignancies and inflammatory diseases [77]. Intensive pharmacological research has focused on the design of BET protein inhibitors (iBETs). One of the earliest developed and best studied is JQ1. This iBET was developed by Jun Qi, a researcher belonging to Chris French's group [78], who demonstrated that JQ1 competes for the bromodomain-binding pocket and displaces BET proteins from the binding to acetylated lysine residues located in histones. This interrupts the remodelling of chromatin and prevents the expression of certain genes [78]. First studies were done in experimental proliferative pathologies, including midline carcinoma and hematological malignances, showing that JQ1 regulates cell proliferation and apoptosis, by reducing the expression of cancer-promoting genes, such as c-Myc and bcl-2, two target genes of BRD4 [79], but now, many preclinical studies have shown its beneficial effects in different pathologies. In a murine model of polycystic kidney disease, iBET JQ1 delayed cyst growth and preserved renal function, by inhibiting c-Myc gene and cystic epithelial cell proliferation [80]. Many *in vitro* studies have described that iBETs could exert anti-inflammatory actions, by inhibiting proinflammatory gene expression [81–83]. Our group had recently described that in cultured tubular epithelial cells stimulated with TNF-*α*, treatment with JQ1 decreased the expression of several genes associated with inflammatory processes and immune response [84]. Moreover, in different models of renal damage (UUO, immune-mediated glomerulonephritis, and angiotensin II-induced renal damage), we showed that JQ1 treatment inhibited renal inflammation [84]. By chromatin immunoprecipitation experiments, we described that the mechanism involved in the anti-inflammatory actions of JQ1 is mediated by the displacement of BRD4 binding to acetylated histone H3 in the promoter region of several proinflammatory genes (such as *IL-6*, *CCL-2*, and *CCL-5*) (Figure 3). Additionally, the role of BRD4 on the regulation of proinflammatory genes was demonstrated by gene silencing [84]. Some preclinical studies have confirmed this anti-inflammatory mechanism of iBETs in other experimental pathologies [85, 86].

BET proteins can also bind to acetylated residues in other proteins besides histones, including transcription factors, regulating gene transcription. In an experimental diabetic model of renal damage in db/db mice, increased acetylation of transcription factors NF-*κ*B and STAT3 were found, showing that modulation of these signalling pathways could be an important mechanism involved in renal inflammation [87]. Many experimental studies have found that blockade of the NF-*κ*B pathway using specific inhibitors of this pathway, or indirectly by drugs used in the clinic to treat CKD patients, including blockers of the renin angiotensin system, attenuates renal inflammation and ameliorates disease progression [88, 89]. The RelA NF-*κ*B subunit is activated by acetylation of lysine 310. Intensive research in the cancer field has unraveled the role of BET proteins in NF-*κ*B pathway regulation, demonstrating that BRD4 binding to acetylated lysine-310 of RelA is essential for recruiting BRD4 and CDK9 to the promoters of specific NF-*κ*B target genes [90–93]. We have described that JQ1 reduced RelA nuclear levels in several models of renal damage and in cultured cells in a proinflammatory environment and thereby blocked NF-*κ*B transcriptional activation and downregulated several NF-*κ*B-controlled genes, including *mcp1* and *il17a* [84], suggesting another mechanism contributing to the anti-inflammatory effects of JQ1 in renal damage. This mechanism has been confirmed with other iBETs [94], associated with inhibition of renal infiltration of macrophages (Figure 3).

Studies blocking Th17 immune response by different approaches, including neutralizing antibodies against IL-17A, soluble receptor, ROR*γ*t inhibitors, or genetically modified mice, have demonstrated the involvement of this immune response in chronic inflammatory diseases, including immune and nonimmune experimental renal damage [95–102]. BET proteins have been involved in the differentiation of naïve CD4 T lymphocytes into Th17 cells [103]. In two models of experimental renal damage, UUO, and immune-mediated glomerulonephritis, we have found that JQ1 treatment markedly diminished the presence of IL-17A-expressing cells and the renal levels of IL-17A and other Th17-related cytokines, such as *ccl20* and *csf1* [84]. Other studies in different pathologies support these data, as described in collagen-induced arthritis and experimental autoimmune encephalomyelitis [104, 105]. BET proteins can modulate Th17 response by a direct effect on *il17a* gene expression. BRD4 and BRD2 bind to the regulatory region of CNS2 that controls IL-17A transcription [99]. Moreover, p300, a transcriptional coactivator that possesses bromodominium and acetylase activity, binds to the promoter of the *il17a* gene in murine Th17 cells, facilitating accessibility to chromatin [106]. In summary, these data expand the neutralization strategies of inflammatory effects mediated by IL-17A in the kidney using iBETs.

Several evidences suggest that iBETs can also present antifibrotic properties, as described in pulmonary fibrosis [104], liver [105], and cardiac damage [107]. The *in vitro* experiments carried out in tubular epithelial cells stimulated with TGF-*β*1 showed that the inhibition of BRD4 functions by silencing their gene or treatment with JQ1 leads to a decrease in the expression of fibrotic genes, such as *α*-smooth muscle actin and fibronectin [108]. In the UUO model, treatment with I-BET151 decreased ECM protein levels and the activation of renal fibroblasts [94]. Recent studies confirm the beneficial effect of JQ1 in fibrosis and inflammation consequent to the radiation used in radiotherapy in thoracic cancer, by suppressing BRD4, c-MYC, collagen I, TGF-*β*, p65, p-SMAD2, and p-SMAD3 after irradiation [109].

Different pharmacological companies have developed several iBETs; some of them are now being used in clinical trials. Most of them have been used in different types of cancer, such as breast cancer, small cell lung cancer, or prostate cancer. For example, ABBV-075 presents a potential antineoplastic activity, leading to an inhibition of cell growth in certain tumors [110]. TEN-010, from Roche, is tested in patients with acute myeloid leukemia [75]. RVX000222, of Resverlogix, was evaluated in patients with diabetes mellitus type 2 and with cardiovascular diseases of high risk [75]. Future studies in renal diseases are required.

#### 1.2.3. Histone Methylation

The process of histone methylation is related to the capacity to transfer one, two, or three methyl groups (CH3) from the S-adenosyl-L-methionine cofactor to lysine or arginine residues in the histone, generating mono-, di-, or trimethyl lysine/arginine residues. This process is developed by a group of enzymes named histone methyltransferases (HMTs) [111]. Some methylation in specific lysine modifications exerts an activating function (H3K4me3) facilitating the opening of the chromatin and the union of the transcriptional machinery, while other modifications are repressive (H3K27me3, H3K9me2/3, and H4K20me3,), allowing the compaction of chromatin and avoiding the binding of the transcriptional machinery [112].

Epigenetic modulation through PTMs such as histone methylation can contribute to renal fibrosis (Figure 1). In the experimental UUO model in mice, H3K9me3 methylation was increased mainly in proximal tubules and myofibroblasts of obstructed kidneys. In the same study, *in vitro* analysis done in primary rat renal fibroblasts and proximal tubule cells (NRK-52e), TGF-*β* stimulation increased H3K9me3 associated to induction of *α*-SMA expression [113]. In rat mesangial cells, TGF-*β* increased profibrotic genes such as Col1*α*1 and PAI-1, associated to increased H3K4me mark levels [114]. A study developed in a podocyte-specific PTIP (a component of an H3K4 methyltransferase complex) knockout mice showed a reduction in H3K4me associated with disease phenotype [115].

Recent studies indicate that H3K4me1/2/3 is essential for the deregulation of key genes in the pathogenesis of DN [116]. In this sense, modifications such as H3K4me and H3K9me1 (associated with promoters of proinflammatory genes) activated the expression of these genes in hyperglycemic conditions [117]. Moreover, RelA NF-*κ*B activation was observed in endothelial cells in high-glucose conditions associated with an increase of H3K4me1 in the p65 promoter region [118, 119]. Accordingly, in an animal model of nephrectomy in db/db mice, increased H3K4me2 renal levels were associated with renal function loss and glomerulosclerosis, and this effect was diminished by treatment with MCP-1/CCL2 antagonist (Spiegelmer mNOX-E36) [120]. In an ischemia-reperfusion injury model, the induction of proinflammatory genes such as TNF-*α* and MCP-1 was related to changes in histone profiles along these genes as H3K4m3 or modification in histone-modifying enzymes (Set1 and BRG1) [121, 122].

#### 1.2.4. Histone Phosphorylation

Phosphorylation occurs predominantly on serine, threonine, and tyrosine side chains or residues through a phosphoester bond formation; this constitutes approximately 86.4%, 11.8%, and 1.8%, respectively, of the human phosphor-proteome [123]. Also, this phosphorylation can occur in less percentage in histidine, lysine, and arginine through phosphoramidate bonds. Histone H2AX phosphorylation (*γ*H2AX) at ser139 was induced by ataxia telangiectasia-mutated (ATM) protein kinase, and is a key marker of DNA damage because is accumulated in DNA to recruit DNA repair complex [124, 125].

Previous studies described the role of *γ*H2AX in oxidative stress [126]. In an I/R renal injury model in mice, injured kidneys presented elevated levels of *γ*H2AX mainly located in tubular epithelial cells [127]. Other studies developed in a model of diabetic nephropathy induced by STZ in Nox1-deficient mice showed lower levels of *γ*H2AX in glomerular and tubular extracts in these KO mice compared to elevated levels in diabetic mice, suggesting the possible role of oxidative stress [128]. These results demonstrated the new role of histone phosphorylation in the pathological process that constitutes renal damage (Figure 1).

#### 1.2.5. Histone Crotonylation

Histone crotonylation is a new posttranscriptional histone modification in lysine residues (Kcr). Crotonate is a short-chain unsaturated carboxylic acid (CH_3_CH=CHCO_2_H) that increases the crotonylation in histones. The original structure and different localization of this histone modification established an apparent variation compared to lysine acetylation (Kac) [129, 130]. This type of modification has the function of activating promoters or potential enhancers. Moreover, histone crotonylation is increased in sex chromosomes and marks testis-specific genes [129]. A recent study established the presence of histone crotonylation in kidney damage after AKI using a mouse model induced by folic acid administration [131]. PGC-1 is a transcription factor governing gene expression of mitochondrial biogenesis and function, with a protective role in renal diseases [132]. Enrichment of histone crotonylation at the *pgc1* gene was found in response to inflammatory cytokines such as TWEAK in tubular cells and in AKI kidney tissue [131]. Crotonate administration to folic acid-injected mice during 48 hours increased renal *pgc1α* and *sirt3* mRNA and decreased *ccl2* mRNA expression indicating that crotonate protects from experimental AKI (Figure 1) [131].

### 1.3. miRNAs and Kidney Diseases

miRNAs are a class of endogenous single-stranded noncoding RNAs with a size within 21-25 nucleotides. miRNAs are involved in posttranscriptional regulation of gene expression by binding to the 3′-untranslated regions (3′-UTRs) and 5′-untranslated regions (5′-UTRs) of the target mRNAs and modulating its degradation [133–137]. These noncoding RNAs are considered as epigenetic modulators due to their capacity of modulating gene expression (Figure 1) [134].

The miRNA levels have been investigated in patients with CKD to elucidate their potential role as biomarkers in CKD progression. A study carried out in a focal segmental glomerulosclerosis (FSG) patient cohort showed increased levels of miR-196a in urine of patients, relating this miRNA with proteinuria and interstitial fibrosis [138]. Furthermore, studies performed in podocytes of FSG patients showed decreased levels of miRNAs that belong to miR-30 related to a protective role of the inhibition of Notch1 and p53 pathways, molecules involved in podocyte injury [139, 140]. Studies carried out in proximal tubular epithelial cells and mesangial cells showed that miRNAs belonging to the let-7 miRNA family are positive regulators of renal fibrosis due to their capacity to upregulate the TGF-*β*1 receptor, increasing the inflammatory TGF-*β*1 pathway [141, 142]. miR-21 has also shown a profibrotic effect diminishing PPAR*α* levels and, consequently, lipid oxidation pathways in wild-type mice in response to kidney injury [143]. In contrast, the miR-29 family has shown antifibrotic effects. In a STZ-induced diabetic model developed in miR-29a transgenic mice, miR-29a reduced glomerular fibrosis and inflammation; these results were confirmed in miR-29 knockdown mice that showed an increase in histone deacetylase activity inducing renal dysfunction through podocyte apoptosis and proteinuria [144, 145]. Furthermore, in the UUO model, miR-29 ultrasound-microbubble-mediated gene transfer reduced renal fibrosis [146]. Another antifibrotic miRNA elucidated in plasma of CKD patients was miR-16 [147].

miRNA-146b-5p has a protective role in reducing IL-6 and IL-8 levels in glomerular mesangial cells [148]. miRNA-663a and miRNA-423-5p have been related with the NF-*κ*B signalling pathway, an important target in the development of lupus nephritis (LN) [149]. Moreover, miR-3201 and miR-1273a are downregulated and associated with endocapillary glomerular inflammation in LN patients [150]. Interestingly, miR-223-3p and miR-93-5p are associated with IL-6 levels in stage 4 CKD patients, while in stage 5 CKD patients these miRNAs have been related with eGFR [151].

The role of miRNAs in AKI has been also investigated, although its contribution to the disease is not well understood. A study carried out in an experimental model of cisplatin-induced AKI in mice showed an antifibrotic role of miR-122 inhibiting FOXO3 translocation and, therefore, reducing inflammatory cell infiltration and increasing cell viability and fibrosis. In the same study, miR-34 was described as profibrotic miRNA since it is involucrate in upregulating foxo3 levels [152]. miR-146a is also related to the inflammatory process in AKI. This miRNA is upregulated in inflammation states through IL-1/TLR activation of NF-*κ*B in cultured renal proximal tubular cells [153]. Other miRNAs have been found to be downregulated in AKI and proposed as protective miRNAs such as miR-17-92, whose overexpression ameliorates I/R injury effects on kidney cells [154]. Additional investigations discovered upregulated miRNAs such as miR-20a, miR-192, miR-187, miR-805, and miR-194 in kidneys of C57BL/6 mice with I/R injury [155].

Different miRNAs have been associated with DN development. miR-192 has been shown to be increased in renal biopsies of diabetic nephropathy patients [156] related to fibrosis and a decreased glomerular filtration rate [157]. miR-192 is upregulated by TGF-*β*, triggered by the increase in glucose levels, and is involucrated in TGF-*β*-induced collagen expression. Other miRNAs have been related to TGF-*β*/glucose increased levels. In a UUO model and tubular epithelial cells, it has been observed that miR-21 is upregulated by elevated TGF-*β*/glucose levels [158–160]. In the same way, miR-214 and miR-21 expressions were upregulated due to TGF-*β* increased levels and induced EMT in rat tubular epithelial cells (NRK52E) [161].

In contrast, high-glucose levels downregulate miR-29a expression in HK2 cells, negatively regulating collagen IV expression [162]. miRNA regulation of the inflammatory process in DN patients is still unclear. miR-146a seems to be protective against DN inflammation in an STZ-induced diabetes model in miR-146a (−/−) mice, increasing renal macrophage infiltration and upregulation of proinflammatory genes such as IL-1*β* and IL-18 [163]. Consequently, with the miR-29a effects in fibrosis, Chen et al. [164] reported in type 2 diabetes in db/db mice anti-inflammatory and antifibrotic functions of miR-29b, with results associated with NF-*κ*B-driven renal inflammation and T-bet/Th1-mediated immune response. In contrast, Guo et al. [165] demonstrated a miR-29c proinflammatory effect in DN carried out by tristetraprolin (TTP), targeting DN patients and immortalized mouse podocytes (MPC5).

In any case, a recent review about miRNA in patients with diabetic kidney disease identified miR-21-5p, miR-29a-3p, miR-126-3p, miR-192-5p, miR-214-3p, and miR-342-3p that are consistently dysregulated among different studies, and bioinformatics analysis revealed that all of them play essential roles in diabetic kidney disease. Finally, a recent meta-analysis from 20 datasets from human and animal studies is aimed at recognizing consistently dysregulated miRNAs in renal fibrosis and identifying 5 upregulated and 2 downregulated miRNA [166]. Thus, miR-1423p, miR-2233p, miR-215p, miR-1425p, and miR-2143p were shown to be consistently upregulated in several reports, whereas miR-29c-3p and miR-200a-3p were downregulated. However, the inclusion of different etiologies, together with the mixture of human and animal samples, can hinder to obtain conclusive results.

## 2. Conclusion

Common mechanisms involved in CKD, including oxidative stress, inflammation, and uremic toxins, can contribute to the renal damage progression by inducing epigenetic modifications, as we have reviewed here. One crucial clinical unmet is the lack of effective CKD treatment, with epigenetic drugs being a source of potential and novel therapeutic options due to the dynamic nature and reversibility of epigenetic modifications. Many preclinical studies are using demethylating agents, HDAC inhibitors, or BET protein inhibitors in experimental renal diseases with successful results. On the other hand, clinical studies have also been developed in patients. HDAC inhibitors such as belinostat, entinostat, vorinostat, and panobinostat and BET protein inhibitors, such as ABBV-075 or TEN-010, have been tested in patients with several tumors, such as lymphoma and myeloma, with beneficial effects whereas these compounds do not have enough effective activity in the treatment of renal tumors. Other IBET RVX000222 has been used with good results in patients with diabetes mellitus type 2 and cardiovascular diseases closely related with CKD. However, future studies in renal diseases are required to gain insight into epigenetic mechanisms associated with the development of renal disease. Body fluids are an accessible source for epigenetic biomarkers. DNA methylation and miRNAs have been studied in patients as biomarkers in CKD progression, e.g., diabetic nephropathy and focal segmental glomerulosclerosis, respectively. All of these epigenetic modifications are a novel field of study to find new targets that can be used as therapeutic approaches and treatments in the diagnostic of CKD and in the monitoring of the progression of renal disease.

In addition, considering the high susceptibility of the genome to epigenetic changes mediated by environmental factors during fetal development, it is of pivotal importance to determine whether the maternal environment can condition epigenetic changes in the fetus associated with the development of renal pathologies during adulthood, generating an important tool for the early detection and prevention of kidney diseases.
